# Noninformative Vision of Body Movements can Enhance Tactile Discrimination

**DOI:** 10.1177/20416695211059203

**Published:** 2022-01-12

**Authors:** Yosuke Suzuishi, Souta Hidaka

**Affiliations:** Department of Psychology, 13024 Rikkyo University; 53347 Japan Society for the Promotion of Science; Department of Psychology, 13024 Rikkyo University

**Keywords:** Haptics/touch, Visuo-haptic interactions, Sense of agency, Sense of ownership

## Abstract

Vision of the body without task cues enhances tactile discrimination performance. This effect has been investigated only with static visual information, although our body usually moves, and dynamic visual and bodily information provides ownership (SoO) and agency (SoA) sensations to body parts. We investigated whether vision of body movements could enhance tactile discrimination performance. Participants observed white dots without any textural information showing lateral hand movements (dynamic condition) or static hands (static condition). For participants experiencing the dynamic condition first, it induced a lower tactile discrimination threshold, as well as a stronger SoO and SoA, compared to the static condition. For participants observing the static condition first, the magnitudes of the enhancement effect in the dynamic condition were positively correlated between the tactile discrimination and SoO/SoA. The enhancement of the dynamic visual information was not observed when the hand shape was not maintained in the scrambled white dot images. Our results suggest that dynamic visual information without task cues can enhance tactile discrimination performance by feeling SoO and SoA only when it maintains bodily information.

## Introduction

Daily, we move our body parts to interact with the environment. Studies on crossmodal interactions demonstrate that our percepts consist of multisensory information (Calvert et al., 2004; Stein, 2012) and establish robust and coherent percepts of the outer world (Ernst & Bülthoff, 2004). Vision of the body without any task cues (noninformative vision) has been reported to enhance tactile discrimination performance for the spatial domain (Haggard, 2006; Kennett et al., 2001; Press et al., 2004) and vibrotactile strength (Harris et al., 2007; Zopf et al., 2011), although impairment of the vibrotactile stimuli has also been reported in the detection and/or discrimination tasks near detection thresholds (Harris et al., 2007; Zopf et al., 2011).

Tactile and visual information of bodily movements contributes substantially to establish bodily sensations, especially the sense of ownership (SoO) and agency (SoA). SoO and SoA represent the feelings “I see my body as my own” and “I can control my moving body parts” (Braun et al., 2018; Gallagher, 2000; Longo et al., 2008b; Tsakiris & Haggard, 2005b). Their perceptual characteristics have been investigated using bodily transfer illusions such as the “rubber hand illusion” (RHI) (e.g., Botvinick and Cohen, 1998), in which we receive spatiotemporally corresponding visual and tactile inputs on a rubber hand and our own hand (out of our sight), respectively. With these multimodal inputs, we feel as though the rubber hand is our own hand. SoO occurs with both active and passive hand movements, whereas SoA occurs with only active hand movements during the RHI (Braun et al., 2014; Kalckert & Ehrsson, 2012). SoO is felt on visually presented fake hands with biological plausibility (Costantini & Haggard, 2007; Ide, 2013; Kalckert & Ehrsson, 2012; Tsakiris & Haggard, 2005a, 2005b), whereas it is not necessary to elicit SoA (Gozli & Brown, 2011; Kalckert & Ehrsson, 2012; Moore & Obhi, 2012). Neuroimaging studies have reported that separate brain regions are associated with SoO (e.g., the midline cortical structures) and SoA (e.g., the presupplementary motor area) (e.g., Tsakiris et al., 2010). Interestingly, SoO and SoA have also shown interaction with each other. For instance, a stronger SoO occurs when SoA is simultaneously evoked in bodily transfer illusions (Braun et al., 2014; Dummer et al., 2009; Kalckert & Ehrsson, 2012; Tsakiris et al., 2010).

Zopf et al. (2011) found that noninformative vision degraded detection performance when eliciting SoO, but enhanced discrimination performance irrespective of feeling SoO. By focusing on relationships among individuals, Longo et al. (2008a) further found that the magnitudes of SoO are positively correlated with the enhancement effect of noninformative vision on tactile orientation discrimination performance during RHI. These findings indicate that feeling SoO can enhance the effects of noninformative vision on tactile discrimination.

As our bodies continuously move in daily life, dynamic noninformative vision can also affect tactile perception. However, to the best of our knowledge, previous findings regarding noninformative vision are limited to static situations. It is also unclear whether SoA can modulate tactile perception. In this study, we investigated whether dynamic noninformative vision influences tactile perception in coordination with SoO and SoA. To purely investigate the effects of dynamic noninformative vision, we presented white dots showing lateral hand movements or static hands without any textural information. The enhancement effects of noninformative vision of hands have consistently been reported for the discrimination of vibrotactile strength (Harris et al., 2007; Zopf et al., 2011). Therefore, we investigated the effects of dynamic noninformative vision of the hand on tactile discrimination performance to vibrotactile stimuli. Based on the effects of static noninformative vision (Haggard, 2006; Kennett et al., 2001; Longo et al., 2008a; Zopf et al., 2011), we expected that dynamic noninformative vision would enhance vibrotactile discrimination performance and that this effect would be positively related to the magnitudes of SoO and/or SoA.

In Experiment 1, we investigated the effects of dynamic noninformative vision by presenting hand movements consisted of white dots moving synchronously with the participants’ hands. We found the enhancement effects of the dynamic noninformative vision on tactile discrimination performance along with higher SoO and SoA. In Experiment 2, we investigated whether the presentation of visual movements synchronized with those of participants’ hands was enough to induce the enhancement effects by presenting visual motion of scrambled white dots without having hand shape. There were no enhancement effects of the dynamic noninformative vision.

## Experiment 1

### Method

**Participant** Twenty university students (19 females; 18–26 years old [M  =  19.95]) participated in Experiment 1. All of them reported having normal or corrected-to-normal vision and normal hearing and touch. They were naïve to the purpose of the experiment, which was duly approved by the local ethics committee of Rikkyo University (reference number: 18–5). Written informed consent was obtained from each participant prior to the experiment.

**Apparatus and stimuli** We applied tactile vibrations (40 Hz, 44.1 dBA) via an amplifier (Eishindenki, ED-PZT01B), an audio interface (Roland, EDIROL FA-66), and a vibrating device (Eishindenki, Attachable Speaker M-PZT-02) on the participants’ right-hand dorsum. Also, we applied multiple vibratory strengths by changing the duration of the stimuli (Gescheider & Joelson, 1983).

**Figure 1.**
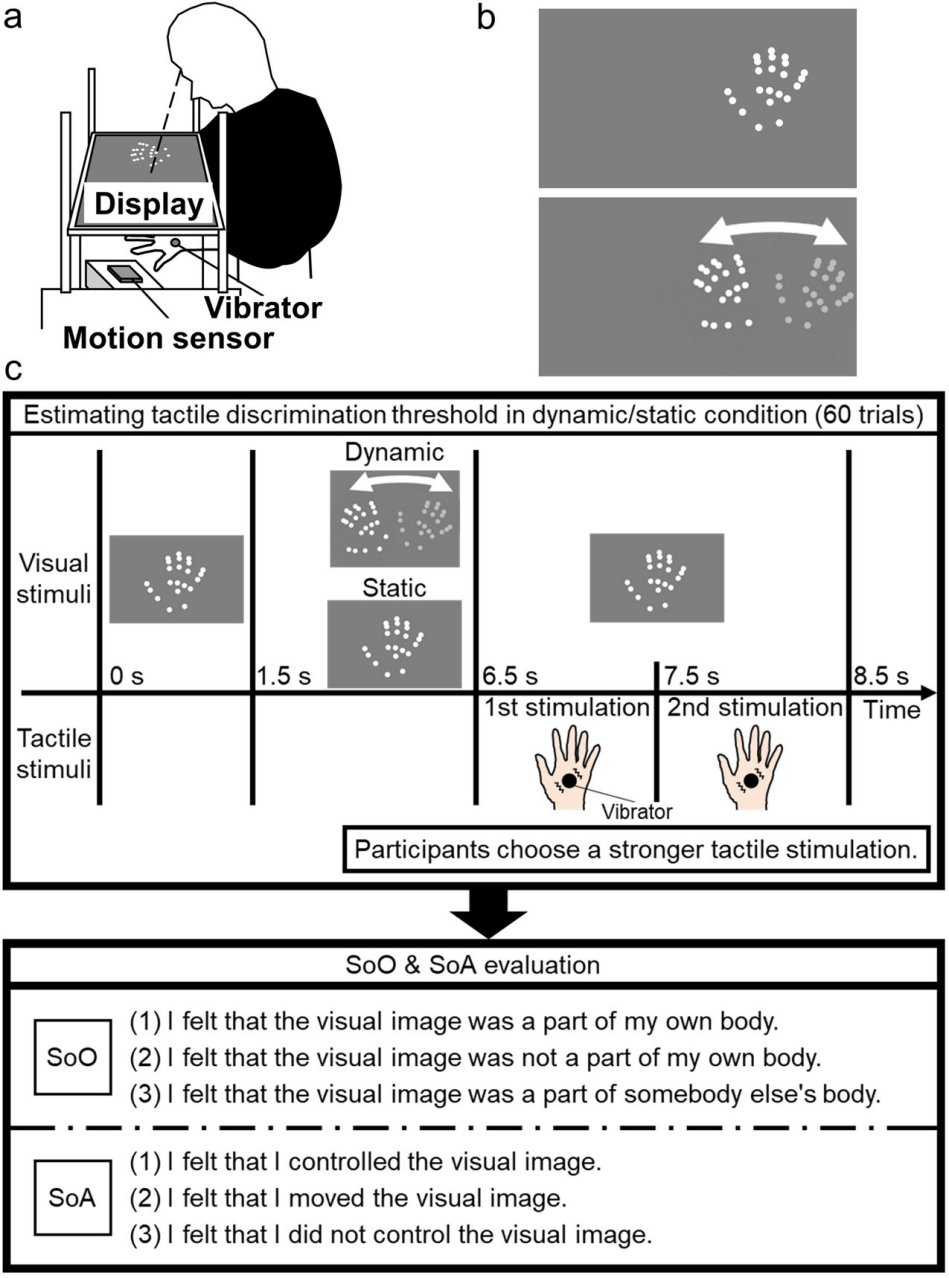
Schematic illustrations of (a) the experimental setups and (b) the visual stimuli for the static and dynamic (upper and lower panels) conditions in Experiment 1. (c) Timeline of each trial in the experimental block and the questionnaires block. The experimental block estimated the tactile discrimination threshold. In the subsequent questionnaires block, SoO and SoA were evaluated for the visual stimulus presented in the experimental block completed just before. The experimental and questionnaires blocks were introduced once for each dynamic and static condition.

The visual stimuli were presented on an LCD (LG, D2342, 1920 × 1080 pixels, 60 Hz) set laterally with a gray background (23.96 cd/m^2^). The viewing distance was 30 cm (Figure 1a). A motion sensor (Leap Motion Controller) tracked the lateral back-and-forth movements of the participants’ right hands. The visual stimuli consisted of 22 white dots (0.51° in radius, 88.69 cd/m^2^) showing the distal interphalangeal, proximal interphalangeal, and metacarpophalangeal joints of each finger, the proximal end of the metacarpal bone of the little finger, and the center of the palm (Figure 1b). These procedures enabled us to present the biological motion (Johansson, 1973, 1976) of hand movements without any textural information. A white dot at the center of the right half of the display served as a landmark indicating the position where participants needed to place their right hand at the start of each trial.

Auditory stimuli (44.8 dBA) were presented through headphones (Pioneer, SE-M531) as cues for trial onset (750 Hz, 1000 ms), hand movements (500 Hz and 1,000 Hz, 100 ms, with 666.67 ms of intervals), and stimulus interval (750 Hz, 50 ms). White noise bursts (37.5 dBA) were also presented during the experiment to prevent participants from hearing artificial noises on tactile stimulations.

The experiment was performed using a customized computer (Dell Precision T3500) and Python with PsychoPy (Peirce, 2007, 2008). Participants responded via foot pedals (Route-R, RI-FP3BK).

**Design and procedure** To investigate whether and how dynamic noninformative vision could affect tactile discrimination performance, we contrasted the dynamic visual information with the static stimuli having an identical shape without any hand movements. Our experiment consisted of experimental and questionnaire blocks (Figure 1c).

Initially, we asked the participants to complete the experimental block in which they performed the tactile discrimination task. In the experimental block, we measured the 82% discrimination threshold of tactile vibratory strength using the QUEST method (Watson & Pelli, 1983). We ran one upward and downward 30-trials staircases, whose durations of the comparison stimulus in the first trial were 50 ms and 200 ms, respectively. The participants wore a black cloth over their arms and hands. They placed their right hand underneath the visual landmark and kept it open. After the trial-onset cue, a dynamic or static condition was introduced. In the dynamic condition, each participant moved their right hand for 4 s according to the hand movement cues. In the static condition, they did not move their hands. Then, with an interval of 1,000 ms to prevent possible tactile suppression of body movements (Juravle et al., 2010, 2017), the standard and comparison vibrotactile stimuli were presented sequentially. The duration of the comparison stimulus was changed based on the participants’ earlier responses, whereas the standard stimulus lasted for 100 ms. We asked the participants to compare the vibratory strength and ignore duration information even when they were aware of the differences in duration. The auditory cues were presented 0–450 ms before each tactile stimulation with a 1,000 ms interval. Participants reported which stimulus felt stronger by pressing the left or right foot pedals (left was first). We confirmed that the perceived strengths of the vibrotactile stimuli changed based on the duration of the stimuli; the stimulus duration was systematically altered in response to the participants’ responses in the QUEST sequences.

Then, in the questionnaire block, the participants were asked to evaluate the subjective magnitudes of SoO and SoA for the visual stimuli presented in the experimental block completed just before using a 7-point Likert scale (-3 (strongly disagree) to + 3 (strongly agree)) with three items (Longo & Haggard, 2009) with minor word modifications (Figure 1c).

The experimental and questionnaire blocks were introduced once for each dynamic and static condition. Each condition included 60 trials in an experimental block (120 trials in total). Each condition lasted 30 min, including the rest, and the duration of the experiment was approximately 60 min in total. Half of the participants completed either the dynamic or static condition first. The order of the standard and comparison stimuli was randomized for each trial in the experimental block. The order of the questionnaires was counterbalanced across the conditions, and that of the items in each questionnaire were randomized for each condition in the questionnaire block. We introduced a practice session under the dynamic condition (10 trials) to familiarize the participants with how to make hand movements in our experimental situation at the beginning of the experiment.

**Analyses** We used JASP (JASP Team, 2020) to perform an analysis of variance (ANOVA). We confirmed the normality of the residuals of the ANOVAs based on the Q-Q plots (Supplementary Figure S1) using Python with bioinfokit (Bedre, 2021) and statsmodels (Seabold & Perktold, 2010). Bayes factor analyses were also performed to estimate how the null or alternative hypotheses were supported. For correlation analyses with the bootstrapped method (10^4^ iterations), we used R (R Core Team, 2020) and RStudio (RStudio Team, 2015) with boot (Canty & Ripley, 2020).

### Results

We found a considerable effect on the order of the experimental conditions (dynamic- or static-condition first) among the participants. Thus, we explicitly evaluated this order effect as a between-subjects factor.

**Figure 2.**
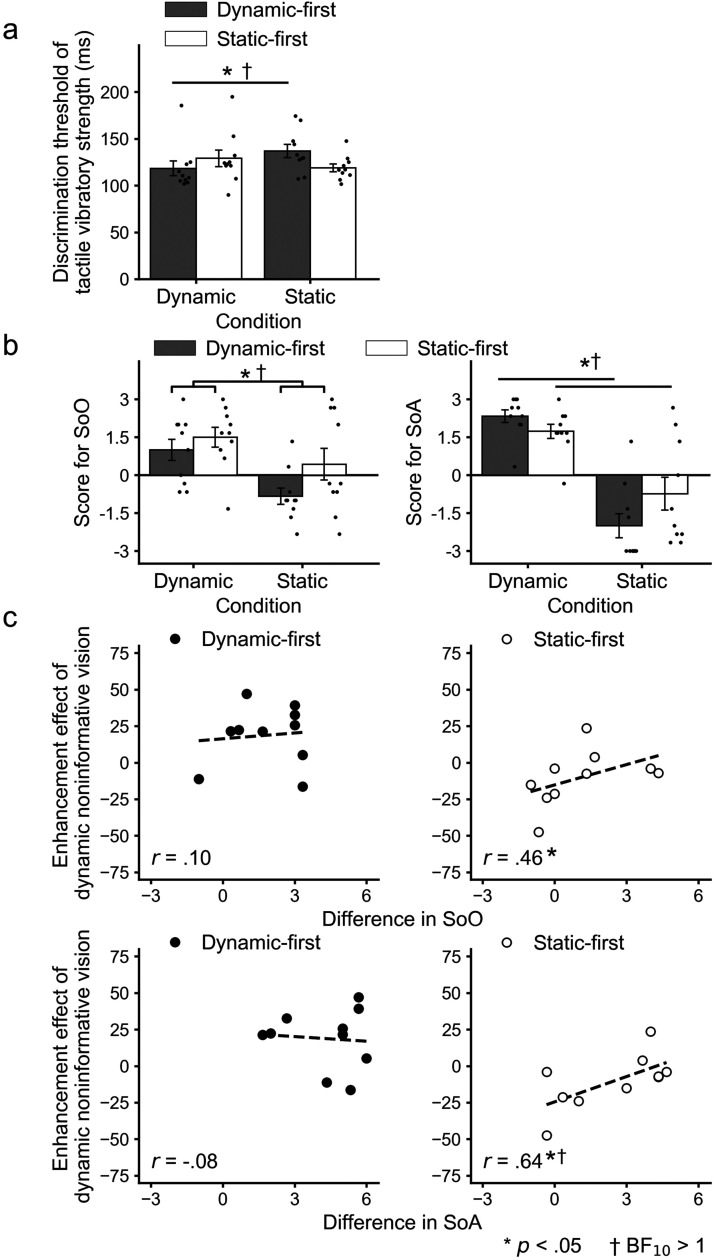
Results of Experiment 1. (a) Discrimination thresholds for the tactile vibratory strength and (b) scores for SoO (left panel) and SoA (right panel) in each experimental condition and each participants’ group. Small dots represent each participant's data, and error bars denote the standard errors of the mean. (c) Scatter plots for the enhancement effect of dynamic noninformative vision between tactile discrimination thresholds and SoO (upper panel) or SoA (lower panel) in each participants’ group. Asterisks indicate statistical significances (*p* < .05), and daggers indicate that Bayes factors support the alternative hypothesis (BF_10_ > 1).

A two-way mixed-design ANOVA with factor condition (dynamic or static) and order (dynamic- or static-first) for the tactile discrimination threshold showed a significant interaction (*F*(1,18)  =  10.92; *p*  =  .004; *η*^2^  =  0.10; BF_10_  =  11.47), whereas no significant main effects of condition (*F*(1, 18)  =  0.91; *p*  =  .35; *η^2^*  =  0.01; BF_01_  =  2.63) and order (*F*s(1,18)  =  0.16; *p*  =  .70; *η^2^*  =  0.01; BF_01_  =  2.15) were observed (Figure 2a). The simple main effect of the condition found that the discrimination threshold in the dynamic condition was lower than that in the static condition in the dynamic-first group (*F*(1, 9)  =  9.07; *p*  =  .007; *η^2^*  =  0.15; BF_10_  =  3.68). In contrast, there was no significant difference in the static-first group (*F*(1, 9)  =  2.76; *p*  =  .11; *η^2^*  =  0.06; BF_10_  =  1.04).

As for the questionnaires, the two-way mixed ANOVA revealed significant main effects of the condition for SoO (*F*(1,18)  =  14.49, *p*  =  .001, *η^2^*  =  0.20, BF_10_  =  54.73) and SoA (*F*(1,18)  =  67.32; *p* < .001; *η^2^*  =  0.59; BF_10_  =  9.84 × 10^6^), showing that they were stronger in dynamic conditions (Figure 2b). The main effect of the order was not significant for SoO (*F*(1,18)  =  2.97; *p*  =  .10; *η^2^*  =  0.08; BF_01_  =  1.19) and SoA (*F*(1,18)  =  0.49; *p*  =  .49; *η^2^*  =  0.01; BF_01_  =  2.62). The interaction effect for SoO was not significant (*F*(1,18)  =  1.01, *p*  =  .33, *η^2^*  =  0.01, BF_01_  =  1.80). The interaction effect was significant for SoA (*F*(1,18)  =  5.07; *p*  =  .004; *η^2^*  =  0.04; BF_10_  =  3.00), but the simple main effects of the condition showed a stronger SoA in the dynamic condition irrespective of the order (*F*s > 30.42, *p*s < .004, *η^2^*s > 0.40, BFs_10_ > 41.99).

We further analyzed the relationships between the effects of dynamic noninformative vision on tactile discrimination thresholds and SoO/SoA among participants. Also, we contrasted the dynamic visual information with static stimuli having an identical shape without any hand movements to investigate the effects of dynamic noninformative vision on tactile discrimination performance. Thus, the index of the enhancement effect was calculated by subtracting the obtained values of the dynamic condition from those of the static condition for each participant. Correlation analyses (*p* < .05/2) showed positive relationships between the differences in the threshold and those in SoA (*r*  =  .46; 97.5% bootstrap CI: .19 < *r* < 1; BF_10_  =  2.18) and SoO (*r*  =  .64; 97.5% bootstrap CI: .02 < *r* < .83; BF_10_  =  0.87) for the static-first group (Figure 2c). In contrast, no significant relationships were found between the differences in the threshold and those in SoO (*r*  =  .10; 97.5% bootstrap CI: - .86 < *r <* 1; BF_01_  =  2.50) nor SoA (*r*  =  - .08; 97.5% bootstrap CI: - .59 < *r <* .58; BF_01_  =  2.53) for the dynamic-first group.

In addition to the main analyses reported above, we also performed correlation analyses between the raw values of the tactile discrimination threshold and those of SoO and SoA for all participants’ data and for each dynamic-first and static-first group (Supplementary Table S1 and Figures S2 and S3). There were no significant correlations between the tactile discrimination threshold and SoO or SoA in both the dynamic and static conditions for all participants and the static-first group. For the dynamic-first group, the tactile discrimination threshold was negatively correlated only with SoO in the dynamic condition. We also performed correlation analyses between the raw values of SoO and SoA (Supplementary Table S1 and Figure S4). There were significant positive correlations between SoO and SoA in the static condition, but not in the dynamic condition, for all participants and the dynamic-first group. For the static-first group, significant positive correlations were found in both the dynamic and static conditions.

## Experiment 2

Experiment 1 showed that dynamic noninformative vision of the hand enhanced tactile discrimination performance along with higher SoO and SoA, although the order effect of the dynamic and static conditions was observed. The question remains whether this effect can be observed simply by the presentation of visual movements synchronized with those of the hand. To evaluate this possibility, we performed an additional experiment with the other participants. We presented dynamic noninformative vision with scrambled white dots images so that the shape of the hand was not maintained.

### Method

The experimental procedures were identical to those used in Experiment 1, except for the following differences.

**Figure 3.**
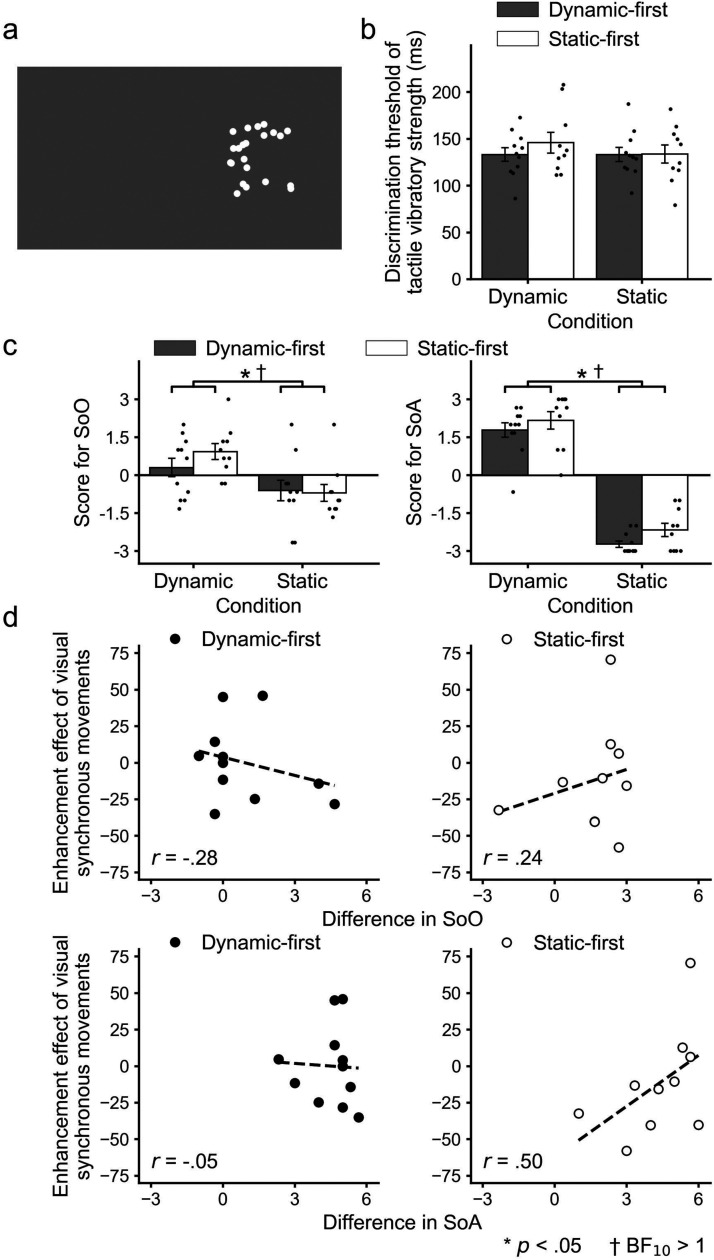
Visual stimuli and results in Experiment 2. (a) An example of the scrambled visual stimulus. (b) Discrimination thresholds for the tactile vibratory strength and (c) scores for SoO (left panel) and SoA (right panel) in each experimental condition and each participants’ group. Small dots represent each participant's data, and error bars denote the standard errors of the mean. (d) Scatter plots for the enhancement effect of dynamic noninformative vision between tactile discrimination thresholds and SoO (upper panel) or SoA (lower panel) in each participants’ group. Asterisks indicate statistical significances (*p* < .05), and daggers indicate that Bayes factors support the alternative hypothesis (BF_10_ > 1).

Twenty-one university students (19 females; 18–26 years old [M  =  20.33]) participated in Experiment 2. The positions of the white dots were randomized for each trial (Figure 3a). In the dynamic condition, the participants were asked to move their right hand laterally. The visual movement of the dots was synchronized with the participants’ hand movements. In the static condition, they were asked to keep their hands still during the tasks. We presented the pre-recorded hand movements of each participant, in which the motion directions of each dot were randomized in each frame. No systematic patterns of visual movement were presented. When we made the recordings, we asked them to practice a few hand movements, without informing them that their movements were being recorded. We recorded the hand movements six times for each participant. Each pre-recorded hand movement was randomly presented five times for each upward and downward staircase in each condition (i.e., each pre-recorded hand movement was presented 20 times in total). We confirmed the normality of the residuals of the ANOVAs based on the Q-Q plots (Supplementary Figure S5).

### Results

The two-way mixed ANOVA with factor conditions (dynamic and static) and order (dynamic- or static-first) for the tactile discrimination thresholds showed no significant main effects of the condition (*F*(1,19)  =  0.77; *p*  =  .39; *η^2^*  =  0.01; BF_01_  =  2.50) or order (*F*(1,19)  =  0.40; *p*  =  .54; *η^2^*  =  0.01; BF_01_  =  2.13), and no significant interaction (*F*(1,19)  =  0.75; *p*  =  .40; *η^2^*  =  0.01; BF_01_  =  1.90) (Figure 3b).

As for the questionnaires, the two-way mixed ANOVA showed significant main effects of the condition both for SoO (*F*(1,19)  =  11.28; *p*  =  .003; *η^2^*  =  0.24; BF_10_  =  62.70) and SoA (*F*(1,19)  =  242.90; *p* < .001; *η^2^*  =  0.87; BF_10_  =  2.51 × 10^17^), whereas neither the main effect of the order (SoO: *F*(1,19)  =  0.62; *p*  =  .44; *η^2^*  =  0.01; BF_01_  =  2.53, SoA: *F*(1,19)  =  3.77; *p*  =  .07; *η^2^*  =  0.01; BF_01_  =  2.65) nor interaction (SoO: *F*(1,19)  =  0.92; *p*  =  .35; *η^2^*  =  0.02; BF_01_  =  1.64, SoA: *F*(1,19)  =  0.10; *p*  =  .75; *η^2^*  =  0.00; BF_01_  =  13.10) was significant (Figure 3c).

There were no significant correlations (*p* < .05/2) for the indices of the enhancement effect (differences between the dynamic and static conditions) between the tactile threshold and SoO/SoA for both the dynamic- (SoO: *r*  =  - .28; 97.5% bootstrap CI: - .99< *r* *<* .37; BF_01_  =  1.96, SoA: *r*  =  - .05; 97.5% bootstrap CI: - .61< *r* *<* .55; BF_01_  =  2.69) and static-first (SoO: *r*  =  .24; 97.5% bootstrap CI: - .61< *r* *<* .55; BF_01_  =  2.13, SoA: *r*  =  .50; 97.5% bootstrap CI: - .02< *r* *<* .97; BF_01_  =  1.00) groups (Figure 3d).

Additional correlation analyses between the raw values of the tactile discrimination threshold and those of SoO/SoA (Supplementary Table S2 and Figures S6 and S7) showed that the tactile discrimination threshold was negatively correlated with SoA in the static condition with no significant correlations for SoO and SoA in the dynamic condition and SoO in the static condition for all participants. For the dynamic-first group, the tactile discrimination threshold did not correlate with the SoO or SoA in both dynamic and static conditions. For the static-first group, the tactile discrimination threshold was negatively correlated with SoO and SoA in the static condition, but there were no significant correlations for SoO and SoA in the dynamic condition. The correlation analyses between the raw values of SoO and SoA (Supplementary Table S2 and Figure S8) revealed that there were positive correlations between SoO and SoA in both dynamic and static conditions for all participants. For the dynamic-first group, SoO positively correlated with SoA in the dynamic condition, but not in the static condition. For the static-first group, SoO positively correlated with SoA in the static condition, but not in the dynamic condition.

## Discussion

This study investigated whether, and how, dynamic noninformative vision modulates tactile discrimination performance in coordination with SoA/SoO. In Experiment 1, we found that dynamic noninformative vision without bodily texture information and task cues enhanced tactile discrimination performance as well as SoO and SoA, although there was a considerable effect of the order of the experimental condition. For the participants exposed to the dynamic condition first, dynamic noninformative vision enhanced tactile discrimination performance at group level. In contrast, the participants who observed static noninformative vision first showed positive correlations between the enhancement of dynamic noninformative vision on tactile discrimination and SoA/SoO among individuals. These enhancement effects of dynamic noninformative vision on tactile discrimination were not observed when the hand shape was not maintained, although dynamic noninformative vision induced greater SoO and SoA (Experiment 2). Thus, our findings cannot be explained simply by presenting visual movements synchronized with the hand movements.

One may consider that the order effect in Experiment 1 can be explained by artificial effects such as fatigue or our experimental manipulation, where we introduced the practice of hand movements at the beginning of the experiment under the dynamic condition. We consider that this was not the case because no order effect was observed in Experiment 2, whose procedures were identical to Experiment 1.

We speculate that the order effect observed in Experiment 1 may have been caused by implicit comparisons of SoO/SoA for noninformative vision. Similar to previous RHI findings (e.g., Kalckert and Ehrsson, 2012), the dynamic visual information synchronized with one's own body movements induced a stronger SoO/SoA compared to the static condition. It is notable that there seem to be larger differences in the magnitudes of SoO and SoA between the dynamic and static conditions for the dynamic-first group compared to the static-first group (Figure 2b). This suggests that the participants who were exposed to the dynamic condition first could feel stronger SoO/SoA for dynamic noninformative vision. Therefore, the enhancement effect of the dynamic noninformative vision may be observed clearly at group level. We should also note, however, that four (SoO) and three (SoA) out of ten participants in the static-first group felt positive sensations even in the static condition for the static-first group (c.f. Kalckert and Ehrsson, 2012). As part of the static-first group could feel the static visual hand image as their own, individual differences may be observed for the enhancement effect of dynamic noninformative vision. Future studies need to evaluate these speculations, for example, by adopting a between-participants design.

To purely investigate the effects of dynamic noninformative vision on tactile discrimination performance, we contrasted the dynamic visual information with the static stimuli having an identical shape. Our main findings were based on the differences between these conditions, which could control possible individual variabilities for tactile discrimination and the feelings of SoO and SoA. Our additional correlation analyses did not show any systematic relationships between the raw values of the tactile discrimination thresholds and those of SoO/SoA and between the raw values of SoO and SoA in both experiments. Future studies need to investigate the commonalities and differences of dynamic and static noninformative vision against other control conditions such as non-body objects (Haggard, 2006).

The current study provides the first demonstration of the enhancement effect of dynamic noninformative vision of the hand in coordination with stronger SoO and SoA. The enhancement effects of dynamic noninformative vision were observed only when the visual stimuli maintained the hand shape. Our findings suggest that dynamic noninformative vision with bodily information facilitates tactile sensation with feelings of SoO and SoA. Static noninformative vision is considered to modulate tactile processing in the primary somatosensory cortex (S1) (Cardini et al., 2012; Fiorio & Haggard, 2005; Haggard et al., 2007; Taylor-Clarke et al., 2002). Additionally, the posterior parietal cortex is assumed to be associated with SoO (Tsakiris, 2010) and SoA (Haggard, 2017). These brain areas, as well as those related to visual and tactile motion processing like hMT + (Hagen et al., 2002), can be involved in the enhancement effect of dynamic noninformative vision on tactile discrimination by feeling SoO and SoA.

Further investigations are necessary in future research to further understand the phenomenological aspects and underlying mechanisms of the enhancement effect of dynamic noninformative vision. Our study presents visual dots to purely investigate the effects of dynamic hand information. Since stronger SoO can be felt for visual images with biological plausibility (Costantini & Haggard, 2007; Ide, 2013; Kalckert & Ehrsson, 2012; Tsakiris & Haggard, 2005a, 2005b), we could expect that the dynamic noninformative vision containing bodily texture as well as bodily shape would induce a stronger enhancement effect. Brain imaging studies also need to be performed to understand the neural mechanisms related to dynamic noninformative vision and neural connections between dynamic noninformative vision and feelings of SoO/SoA.

## Supplemental Material

sj-docx-1-ipe-10.1177_20416695211059203 - Supplemental material for Noninformative Vision of Body Movements can Enhance Tactile Discrimination Click here for additional data file. Supplemental material, sj-docx-1-ipe-10.1177_20416695211059203 for Noninformative Vision of Body Movements can Enhance Tactile Discrimination by Yosuke Suzuishi and Souta Hidaka in i-Perception
