# Supplementary material for: Noninformative Vision of Body Movements can Enhance Tactile Discrimination
Source: Iperception. 2022 Jan 12;13(1):20416695211059203. doi: 10.1177/20416695211059203 (PMC8761889; doi:10.1177/20416695211059203)
Supplement: sj-docx-1-ipe-10.1177_20416695211059203 - Supplemental material for Noninformative Vision of Body Movements can Enhance Tactile Discrimination [file sj-docx-1-ipe-10.1177_20416695211059203.docx]

## Supplementary information

**Noninformative vision of body movements can enhance tactile discrimination**

Yosuke Suzuishi^1,2^ & Souta Hidaka.^1^

1. Department of Psychology, Rikkyo University

2. Japan Society for the Promotion of Science

*Corresponding author:

Yosuke Suzuishi

E-mail: suzuishi@rikkyo.ac.jp

Address: Department of Psychology, Rikkyo University, 1-2-26, Kitano, Niiza-shi, Saitama, 352-8558 Japan.

## Supplementary Tables.

**Supplementary Table S1.** Results of the correlation analyses across the raw values of the tactile discrimination threshold, SoO, and SoA in Experiment 1.

|  |  | All participants | | | | |
| --- | --- | --- | --- | --- | --- | --- |
|  | Dynamic | | | Static | | |
|  | *r* | 97.5% CI | BF_10_ | *r* | 97.5% CI | BF_10_ |
| SoO × Threshold | - .47 | - 1 < *r* < .08 | 2.05 | - .20 | - .67 < *r* < .25 | 0.33 |
| SoA × Threshold | - .28 | - 1 < *r* < .47 | 0.53 | - .08 | - .58 < *r* < .35 | 0.29 |
| SoO × SoA | .45 | - .10 < *r* < 1 | 1.71 | **.81** | **.59 < *r* < 1** | **1714.27** |
|  | Dynamic-first participants | | | | | |
|  | Dynamic | | | Static | | |
|  | *r* | 98.75% CI | BF_10_ | *r* | 98.75% CI | BF_10_ |
| SoO × Threshold | **- .54** | **- 1 < *r* < - .05** | **1.23** | .35 | - .55 < *r* < 1 | 0.61 |
| SoA × Threshold | .26 | - .43 < *r* < 1 | 0.49 | .14 | - .63 < *r* < .81 | 0.41 |
| SoO × SoA | .29 | - .43 < *r* < 1 | 0.52 | **.80** | **.39 < *r* < 1** | **11.46** |
|  | Static-first participants | | | | | |
|  | Dynamic | | | Static | | |
|  | *r* | 98.75% CI | BF_10_ | *r* | 98.75% CI | BF_10_ |
| SoO × Threshold | - .53 | - 1 < *r* < .61 | 1.15 | - .36 | - 1 < *r* < .38 | 0.62 |
| SoA × Threshold | - .61 | - 1 < *r* < .38 | 1.85 | - .05 | - .90 < *r* < .93 | 0.39 |
| SoO × SoA | **.84** | **.25 < *r* < 1** | **21.34** | **.79** | **.42 < *r* < 1** | **9.36** |

# Bold letters indicate the results with statistical significance (*p* < .05).

**Supplementary Table S2.** Results of the correlation analyses across the raw values of tactile discrimination threshold, SoO, and SoA in Experiment 2.

|  |  | All participants | | | | |
| --- | --- | --- | --- | --- | --- | --- |
|  | Dynamic | | | Static | | |
|  | *r* | 97.5% CI | BF_10_ | *r* | 97.5% CI | BF_10_ |
| SoO × Threshold | .15 | - .19 < *r* < .49 | 0.33 | - .35 | - .94 < *r* < .26 | 0.82 |
| SoA × Threshold | .22 | - .28 < *r* < .72 | 0.41 | **- .47** | **- .85 < *r* < - .12** | **2.27** |
| SoO × SoA | **.63** | **.36 < *r* < .92** | **21.34** | **.43** | **.00 < *r* < .89** | **1.61** |
|  | Dynamic-first participants | | | | | |
|  | Dynamic | | | Static | | |
|  | *r* | 98.75% CI | BF_10_ | *r* | 98.75% CI | BF_10_ |
| SoO × Threshold | .14 | - .59 < *r* < .84 | 0.40 | .05 | - .92 < *r* < 1 | 0.37 |
| SoA × Threshold | .24 | - .34 < *r* < .89 | 0.47 | - .02 | - .73 < *r* < .65 | 0.37 |
| SoO × SoA | **.67** | **.34 < *r* < .98** | **3.58** | .36 | - .38 < *r* < 1 | 0.63 |
|  | Static-first participants | | | | | |
|  | Dynamic | | | Static | | |
|  | *r* | 98.75% CI | BF_10_ | *r* | 98.75% CI | BF_10_ |
| SoO × Threshold | .07 | - .69 < *r* < .79 | 0.39 | **- .83** | **- 1 < *r* < - .63** | **17.15** |
| SoA × Threshold | .14 | - .84 < *r* < 1 | 0.42 | **- .76** | **- 1 < *r* < - .45** | **7.06** |
| SoO × SoA | .56 | - .06 < *r* < 1 | 1.35 | **.68** | **.25 < *r* < 1** | **2.96** |

# Bold letters indicate the results with statistical significance (*p* < .05).

## Supplementary Figures.


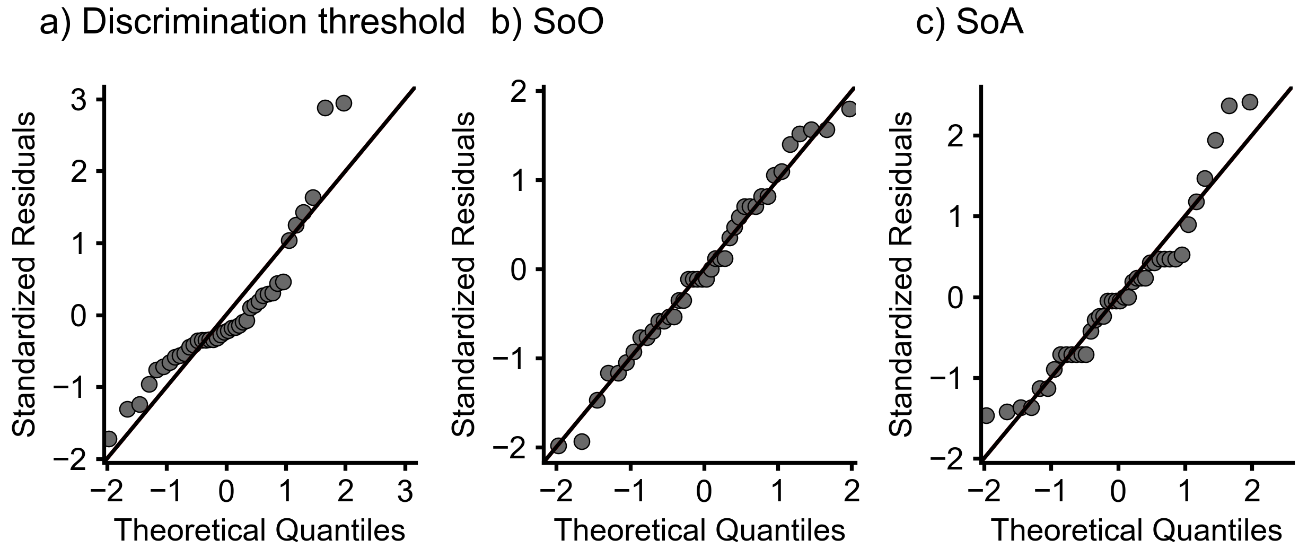


**Supplementary Figure S1.** Q-Q plots of the two-way mixed-design ANOVA with factor condition (dynamic or static) and order (dynamic- or static-first) for (a) tactile discrimination threshold, (b) SoO, and (c) SoA in Experiment 1.


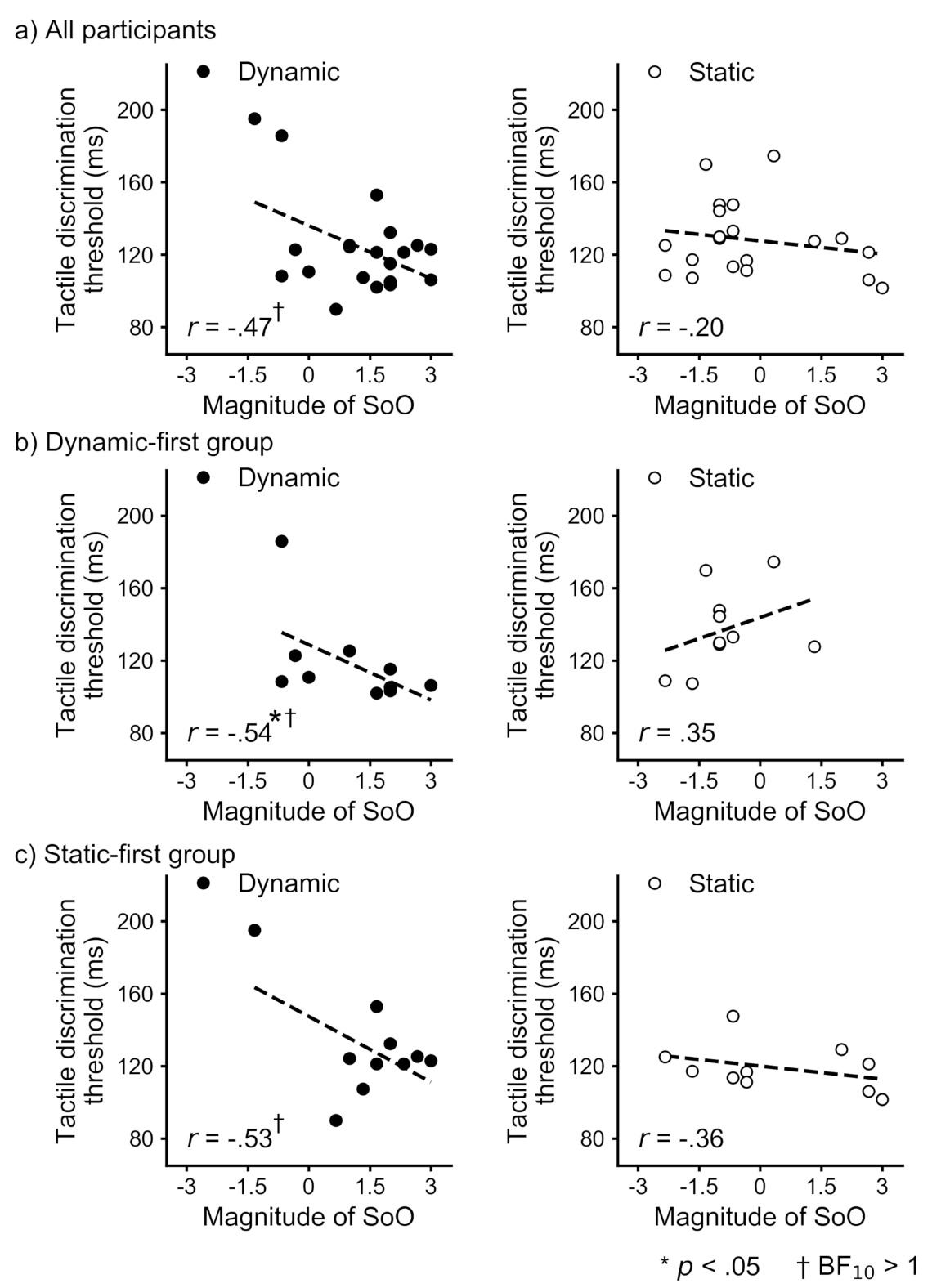


**Supplementary Figure S2.** Scatter plots for the raw values of tactile discrimination threshold and SoO in Experiment 1 as follows: (a) all participants, (b) dynamic-first, and (c) static-first groups. Right and left panels show the dynamic and static conditions, respectively. An asterisk indicates statistical significance (*p* < .05), and daggers indicate that Bayes factors support the alternative hypothesis (BF_10_ > 1).


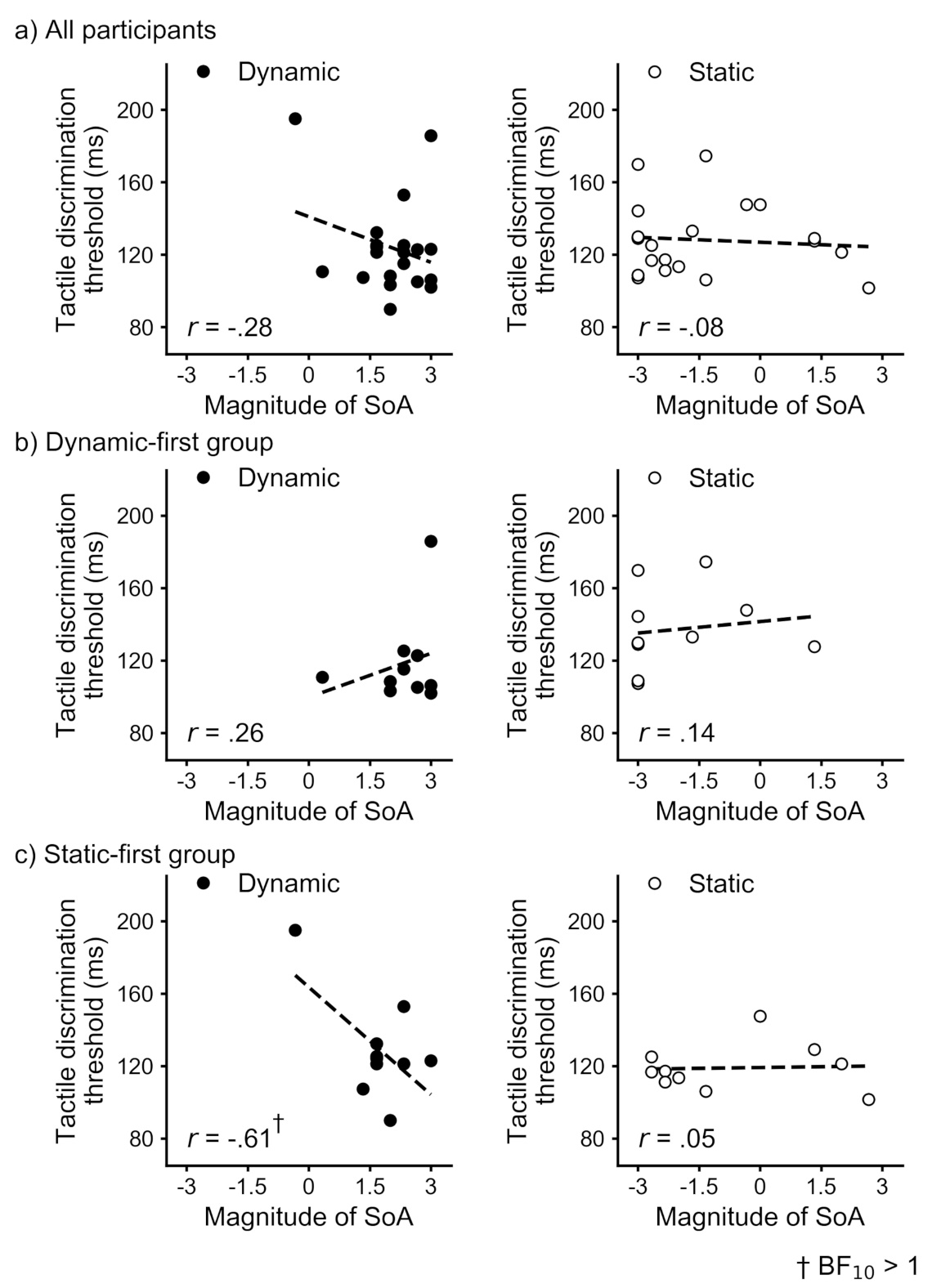


**Supplementary Figure S3.** Scatter plots for the raw values of tactile discrimination threshold and SoA in Experiment 1 as follows: (a) all participants, (b) dynamic-first, and (c) static-first groups. Right and left panels show the dynamic and static conditions, respectively. A dagger indicates that Bayes factors support the alternative hypothesis (BF_10_ > 1).


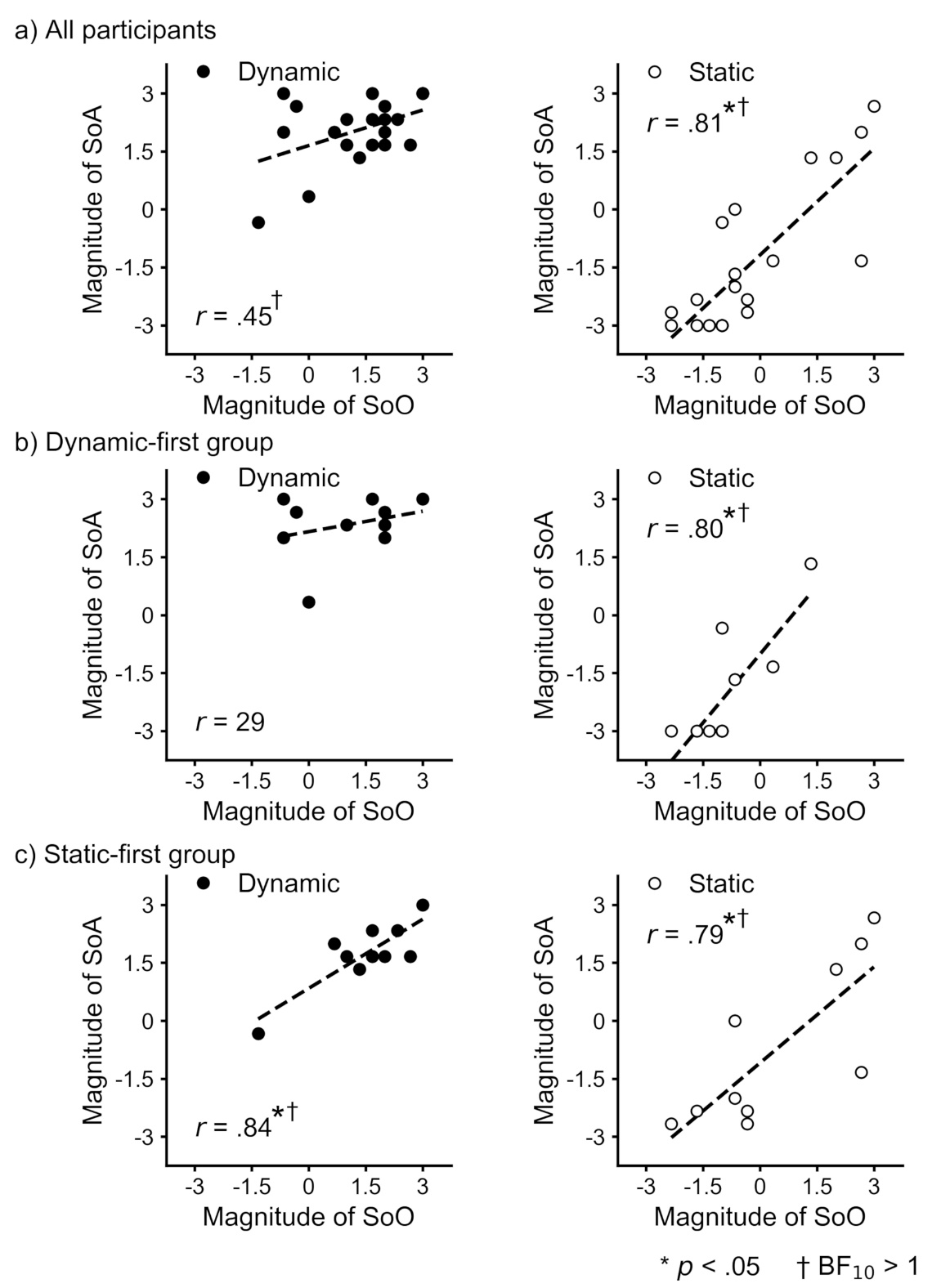


**Supplementary Figure S4.** Scatter plots for the raw values of SoO and SoA in Experiment 1 as follows: (a) all participants and (b) dynamic-first and (c) static-first groups. Right and left panels show the dynamic and static conditions, respectively. Asterisks indicate statistical significance (*p* < .05), and daggers indicate that Bayes factors support the alternative hypothesis (BF_10_ > 1).


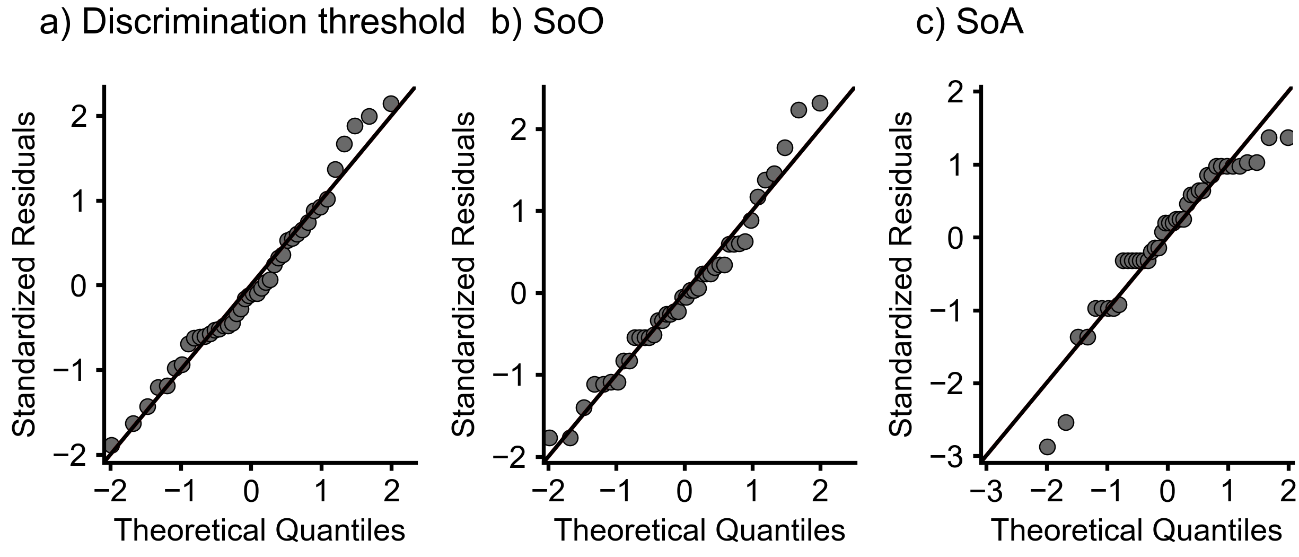


**Supplementary Figure S5.** Q-Q plots of the two-way mixed-design ANOVA with factor condition (dynamic or static) and order (dynamic- or static-first) for (a) tactile discrimination threshold, (b) SoO, and (c) SoA in Experiment 2.


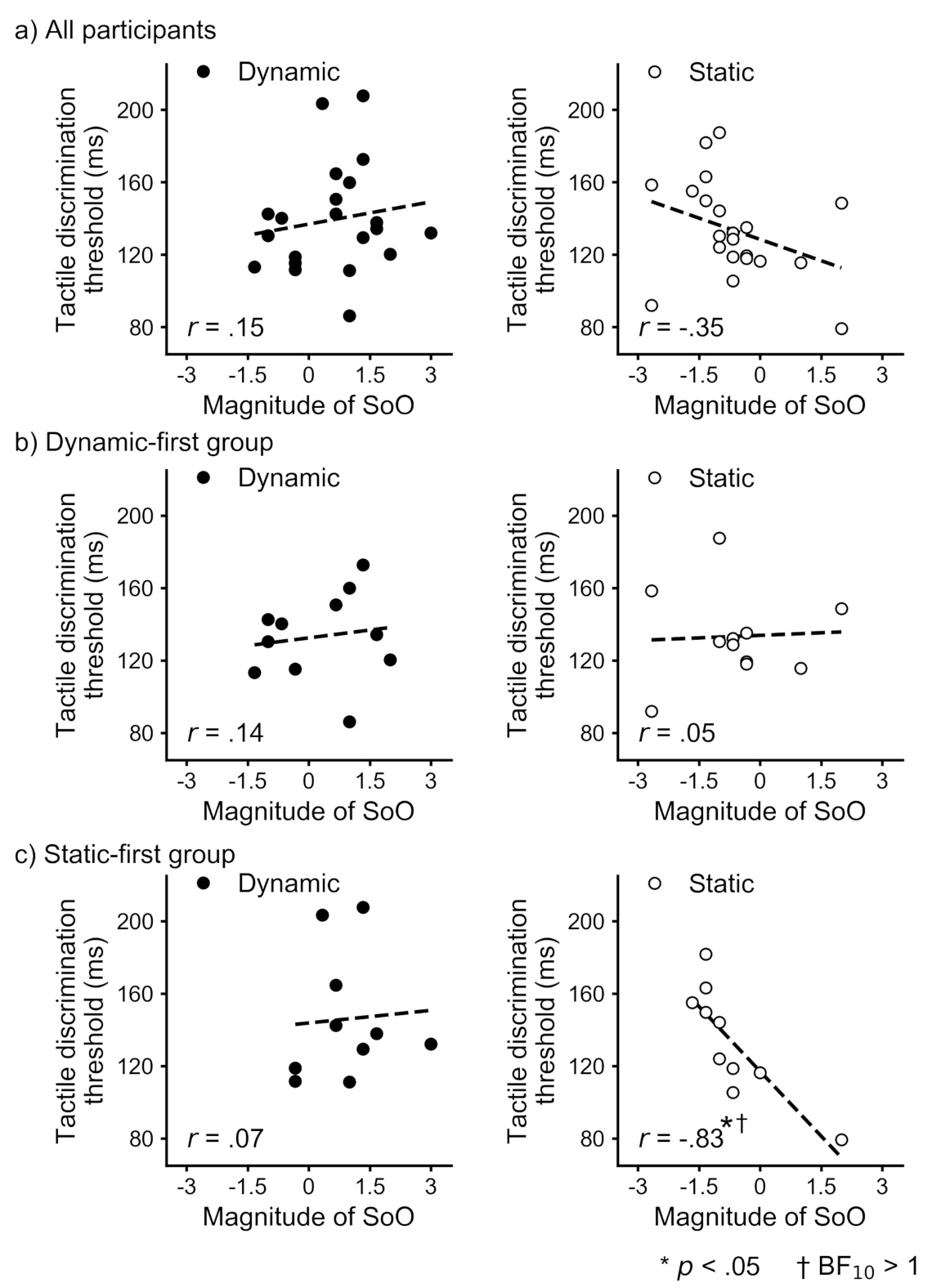


**Supplementary Figure S6.** Scatter plots for the raw values of tactile discrimination threshold and SoO in Experiment 2 as follows: (a) all participants, (b) dynamic-first, and (c) static-first groups. Right and left panels show the dynamic and static conditions, respectively. An asterisk indicates statistical significance (*p* < .05), and a dagger indicates that Bayes factors support the alternative hypothesis (BF_10_ > 1).


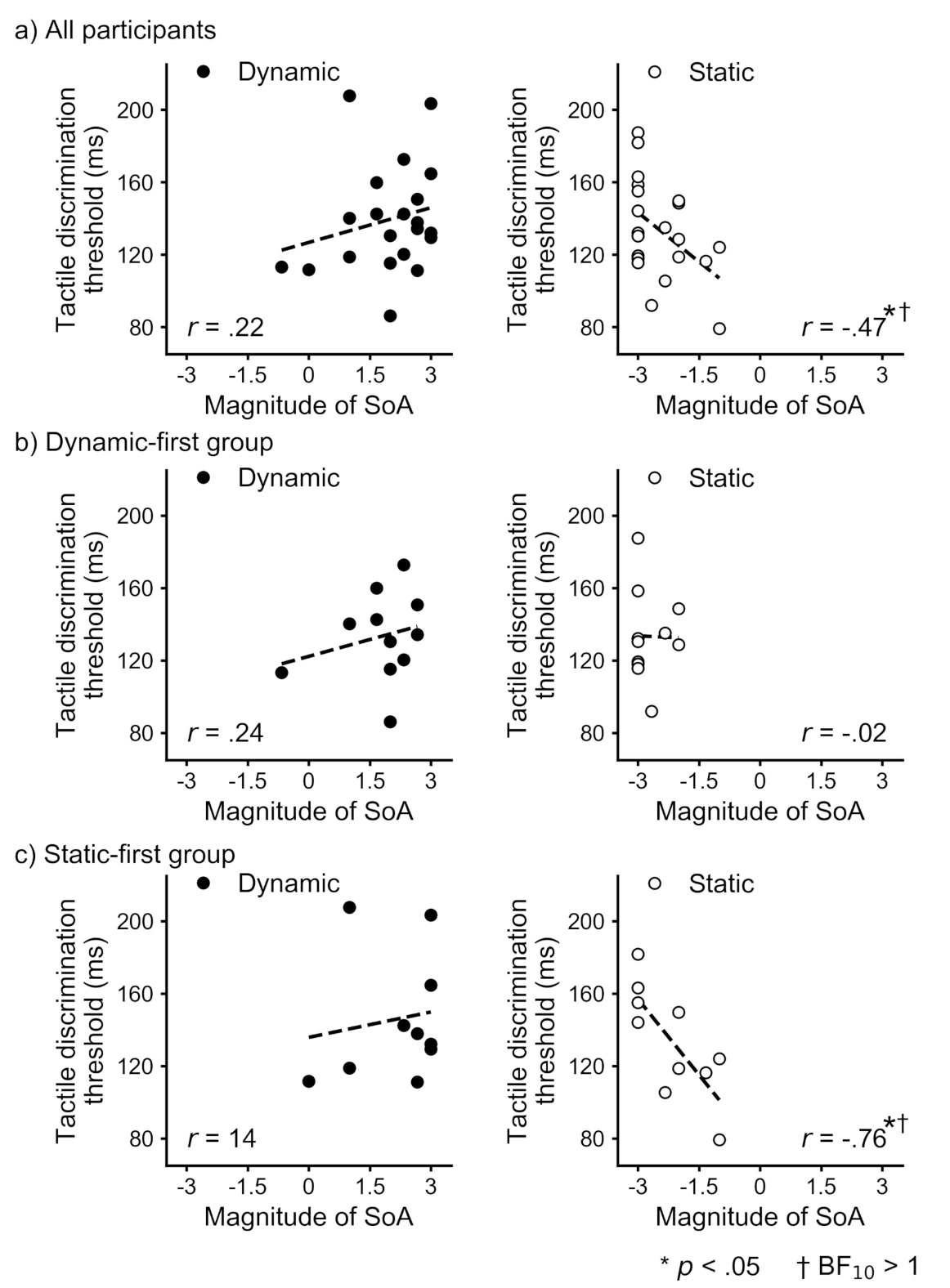


**Supplementary Figure S7.** Scatter plots for the raw values of tactile discrimination threshold and SoA in Experiment 2 as follows: (a) all participants and (b) dynamic-first and (c) static-first groups. Right and left panels show the dynamic and static conditions, respectively. Asterisks indicate statistical significance (*p* < .05), and daggers indicate that Bayes factors support the alternative hypothesis (BF_10_ > 1).


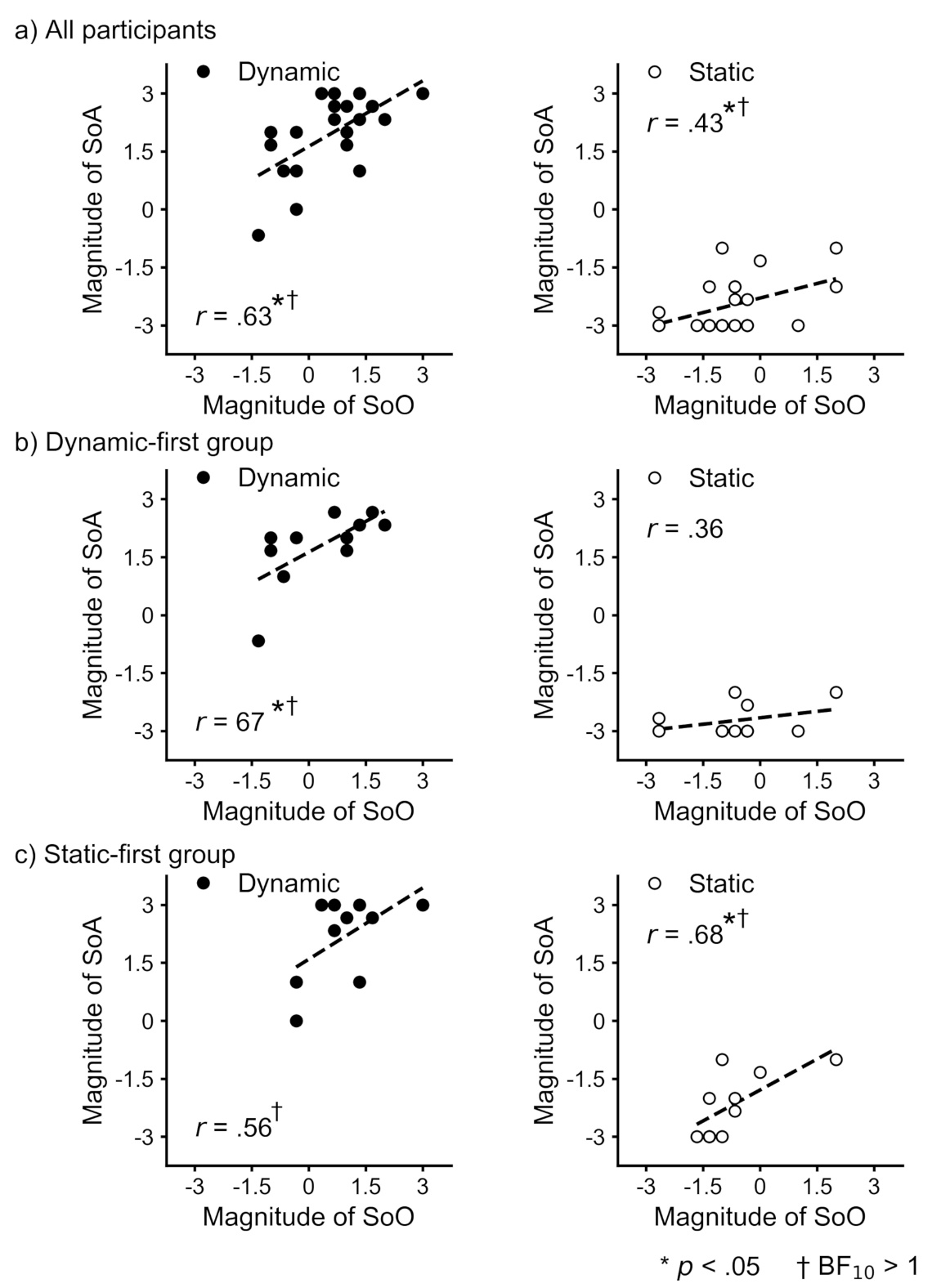


**Supplementary Figure S8.** Scatter plots for the raw values of SoO and SoA in Experiment 2 as follows: (a) all participants and (b) dynamic-first and (c) static-first groups. Right and left panels show the dynamic and static conditions, respectively. Asterisks indicate statistical significance (*p* < .05), and daggers indicate that Bayes factors support the alternative hypothesis (BF_10_ > 1).
